# Impact of maternal education about complementary feeding and provision of complementary foods on child growth in developing countries

**DOI:** 10.1186/1471-2458-11-S3-S25

**Published:** 2011-04-13

**Authors:** Aamer Imdad, Mohammad Yawar Yakoob, Zulfiqar A Bhutta

**Affiliations:** 1 Division of Women & Child Health, The Aga Khan University, Karachi, Pakistan

## Abstract

**Background:**

Childhood undernutrition is prevalent in low and middle income countries. It is an important indirect cause of child mortality in these countries. According to an estimate, stunting (height for age Z score < -2) and wasting (weight for height Z score < -2) along with intrauterine growth restriction are responsible for about 2.1 million deaths worldwide in children < 5 years of age. This comprises 21 % of all deaths in this age group worldwide. The incidence of stunting is the highest in the first two years of life especially after six months of life when exclusive breastfeeding alone cannot fulfill the energy needs of a rapidly growing child. Complementary feeding for an infant refers to timely introduction of safe and nutritional foods in addition to breast-feeding (BF) i.e. clean and nutritionally rich additional foods introduced at about six months of infant age. Complementary feeding strategies encompass a wide variety of interventions designed to improve not only the quality and quantity of these foods but also improve the feeding behaviors. In this review, we evaluated the effectiveness of two most commonly applied strategies of complementary feeding i.e. timely provision of appropriate complementary foods (± nutritional counseling) and education to mothers about practices of complementary feeding on growth. Recommendations have been made for input to the Lives Saved Tool (LiST) model by following standardized guidelines developed by Child Health Epidemiology Reference Group (CHERG).

**Methods:**

We conducted a systematic review of published randomized and quasi-randomized trials on PubMed, Cochrane Library and WHO regional databases. The included studies were abstracted and graded according to study design, limitations, intervention details and outcome effects. The primary outcomes were change in weight and height during the study period among children 6-24 months of age. We hypothesized that provision of complementary food and education of mother about complementary food would significantly improve the nutritional status of the children in the intervention group compared to control. Meta-analyses were generated for change in weight and height by two methods. In the first instance, we pooled the results to get weighted mean difference (WMD) which helps to pool studies with different units of measurement and that of different duration. A second meta-analysis was conducted to get a pooled estimate in terms of actual increase in weight (kg) and length (cm) in relation to the intervention, for input into the LiST model.

**Results:**

After screening 3795 titles, we selected 17 studies for inclusion in the review. The included studies evaluated the impact of provision of complementary foods (±nutritional counseling) and of nutritional counseling alone. Both these interventions were found to result in a significant increase in weight [WMD 0.34 SD, 95% CI 0.11 – 0.56 and 0.30 SD, 95 % CI 0.05-0.54 respectively) and linear growth [WMD 0.26 SD, 95 % CI 0.08-0.43 and 0.21 SD, 95 % CI 0.01-0.41 respectively]. Pooled results for actual increase in weight in kilograms and length in centimeters showed that provision of appropriate complementary foods (±nutritional counseling) resulted in an extra gain of 0.25kg (±0.18) in weight and 0.54 cm (±0.38) in height in children aged 6-24 months. The overall quality grades for these estimates were that of ‘moderate’ level. These estimates have been recommended for inclusion in the Lives Saved Tool (LiST) model. Education of mother about complementary feeding led to an extra weight gain of 0.30 kg (±0.26) and a gain of 0.49 cm (±0.50) in height in the intervention group compared to control. These estimates had been recommended for inclusion in the LiST model with an overall quality grade assessment of ‘moderate’ level.

**Conclusion:**

Provision of appropriate complementary food, with or without nutritional education, and maternal nutritional counseling alone lead to significant increase in weight and height in children 6-24 months of age. These interventions can significantly reduce the risk of stunting in developing countries and are recommended for inclusion in the LiST tool.

## Background

Childhood malnutrition is prevalent in low and middle income countries. According to an estimate, 20 % of children < 5 years of age in these countries were underweight (weight for age Z score < -2) in year 2005 [1]. Similarly, about 32 % of children < 5 years of age in these countries were stunted (height for age Z score < -2). The prevalence of both underweight and stunting was highest in Africa and South-Central Asia. Stunting and wasting (weight for height Z score < -2) along with intrauterine growth restriction are responsible for about 2.1 million deaths worldwide in children < 5 years of age. This comprises 21 % of all deaths in this age group worldwide [1]. It is well recognized that the period of 6-24 months of age is one of the most critical time periods in the growth of the infant. The incidence of stunting is the highest in this period as children have high demand for nutrients and there are limitations in the quality and quantity of available foods, especially after exclusive breastfeeding [2,3].

Complementary feeding for infants refers to the timely introduction of safe and nutritional foods in addition to breast-feeding (BF) i.e. clean and nutritionally rich additional foods introduced at about six months of infant age. These foods are typically provided to children from 6 to 18-24 months of age [4]. It has been suggested that in addition to disease prevention strategies, complementary feeding interventions targeting this “critical window” are most efficient in reducing malnutrition and promoting adequate growth and development [5]. According to the World Health Organization (WHO), the complementary feeding should be *timely*, meaning that all infants should start receiving foods in addition to breastmilk from 6 months onwards; *adequate*, meaning that the nutritional value of complementary foods should fulfill the needs of rapidly growing child; and *appropriate*, meaning that foods should be diverse, of appropriate texture and given in sufficient quantity [4].

Several strategies have been employed to improve complementary feeding practices [3]. These include nutritional counseling to mothers designed to promote healthy feeding practices, provision of complementary foods and supplementation with foods either fortified with multiple micronutrients or with increased energy content [3,6]. A recent review published by Dewey et al. showed that educational interventions for complementary feeding had a modest effect on weight [Weighed mean difference (WMD) = 0.28 SD; range -0.06 to 0.96] and linear growth [WMD = 0.20 SD; range 0.04 to 0.64] [3]. Pooled results for studies where provision of complementary food was the main intervention showed that it had an impact on weight [WMD 0.60 SD; range -0.02 to 2.29] and linear growth [WMD 0.47 SD; range -0.04 to 1.81], however the pooled results were not statistically significant. The review also showed that impact of complementary feeding was enhanced if provision of food was combined with education to mothers. Another review and model simulation published in Lancet Under-nutrition Series demonstrated that provision of complementary food (±nutritional counseling) was associated with a significant increase in linear growth especially in food insecure populations [7].

Because there is no single universal package of components in which all possible effective complementary feeding interventions (mentioned above) are integrated, it is difficult to generalize the impact of efforts to improve complementary feeding [3]. We here evaluate the two most important and commonly used complementary feeding interventions i.e. nutrition counseling alone and provision of complementary foods (with and without counseling) from 6 – 24 months of age in children in developing countries with the aim to obtain a point estimate of the effectiveness of these strategies for input to the Lives Saved Tool (LiST) model. We also assessed/graded available evidence using standardized guidelines of the Child Health Epidemiology Reference Group (CHERG) adaptation of GRADE technique [8,9]. The quantitative measures were based on pooled weighted mean difference (effect size) and pooled mean difference i.e. actual mean change in weight (kg) and length (cm).

## Methods

### Search strategy

**Figure 1:**
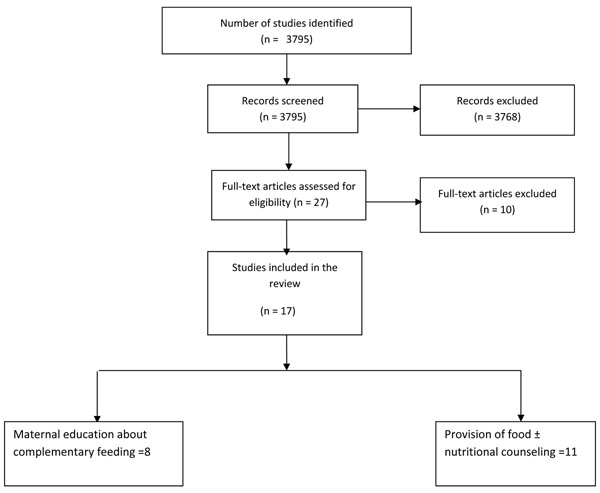
Flow diagram showing identification of studies

To evaluate the impact of complementary feeding interventions on child growth, a search was conducted on PubMed, Cochrane Library and WHO regional databases. The last date of search was 3^rd^ February, 2010. The following search strategy was used: (Child* OR "Child"[Mesh] OR "Child, Preschool"[Mesh] OR Infant* OR "Infant"[Mesh]) AND ("Complementary food*" OR "Complementary Feed*" OR "Supplementary food*" OR "Supplementary feed*" OR "Food, Fortified*"[Mesh] OR "Fortified food" OR "Infant Food"[Mesh]). The search was limited to humans, clinical trial, review and meta-analysis. From the search, relevant titles and abstracts were identified after excluding those that were obviously irrelevant. Full texts of the remaining papers were reviewed to identify studies reporting the outcomes of interest. We also reviewed the reference lists of identified articles, existing reviews and meta-analyses, and looked for studies that were not picked up in the main search. Authors were contacted for any additional data, if required. Figure 1 shows a flow diagram that presents the studies identified, screened for relevance, and final articles where data were extracted.

### Inclusion/exclusion criteria

Complementary feeding interventions evaluated in this review included the following two strategies: a) provision of complementary food with/without nutritional counseling to mothers vs. no intervention, and b) nutritional counseling alone vs. no intervention. Only those studies were included in which provision of complementary food (±education) or educational intervention was the only difference between the two study groups. Studies addressing supplementation of food after 24 months of age were excluded from the review. All the included studies were from developing countries. The developing countries were defined as countries with Gross National Income per capita (GNI) per capita below US$11,905, according to World Bank[10]. The primary outcomes were mean *change* in weight (WAZ scores or kg) and *change* in height (HAZ score or cm). Studies with insufficient data on change in weight or height were excluded from the meta-analysis. All the included studies were randomized (individual or cluster) or quasi-experimental trials. We excluded studies comparing fortified foods with non-fortified foods and those that added multiple micronutrient supplements or powders to complementary foods. Even though we included terms like ‘supplementary food’ and ‘supplementary feed’ in our literature search but only those studies were included where the term supplementary food was used for introduction of additional food to a breastfed child at the age of 6 months i.e. complementary feeding.

### Data abstraction

We abstracted the data into a standardized form [9] that included variables on study context, design and primary outcomes. The specific data extracted on study design included evaluation of adequacy of methods of randomization (sequence generation), allocation concealment, blinding and dealing with missing data. Data related to intervention specifics (dose, duration etc) and study population were also abstracted. Two independent authors entered the data and discrepancies were removed, if found. In the studies where provision of complementary food was the main intervention and they also included children < 6 months of age, disaggregated was abstracted for age group > 6 months only if available.

### Grading of evidence

The available evidence was graded according to the Child Health Epidemiology Reference Group (CHERG) adaptation of the GRADE technique [8]. This method of quality assessment involved a two-step evaluation of evidence i.e. at individual study level and that for overall pooled results. Individual studies were evaluated based on study design, quality of methods, relevance to study population (middle/lower income countries) and directness of outcomes of interests compared with that of other studies. Each study was assigned a final quality grade of “high” “moderate” “low” or “very low” on the basis of strengths and limitations of the study. Any study with a final grade of ‘very low’ was excluded from the analysis.

The grading of overall evidence was based on three components: (1) the volume and consistency of the evidence; (2) the size of the pooled effect and (3) the strength of the statistical evidence reflected in the p-value. A similar grading of ‘high’ ‘moderate’ ‘low’ and ‘very low’ was used for grading the overall evidence indicating the strength of an effect of the intervention on specific health outcome.

### Quantitative data synthesis

We conducted a meta-analysis for an outcome where data were available from more than one study. The main outcomes of interest were impact of complementary feeding interventions on mean *change* in weight (WAZ scores or kg) and length (HAZ scores or cm). The studies where mean change in weight or height was not reported in the study, it was calculated as the difference of mean post- and pre-intervention measurements. If studies did not report the standard deviation (SD) for change, it was calculated assuming that the correlation between the pre- and post-test variances was equal to the average correlation found in available studies. In studies with factorial design, only those data sets were considered where the intervention evaluated (i.e. provision of food or nutritional counseling) was the only difference between the intervention and comparison group. In case of cluster design, cluster adjusted values were used.

We first pooled the data to get a standardized weighted mean difference (WMD) also called ‘standard mean difference’ or ‘effect size’. The advantage of calculating WMD is that it eliminates the problems of units of measurement and duration, which may vary across studies [11]. For example, data can be pooled using change in Z score for length or actual change in mean length in centimeters to get a pooled WMD. The formula for calculating effect size is

Results of effect size are interpreted as the percent of non-overlap of the intervention group’s scores with those of the control group. An effect size of 0.0 indicates that the distribution of scores for the intervention group overlaps completely with the distribution of scores for the control group, and there is 0% non-overlap. A weighed mean difference (WMD)of 0.2 represents a small effect, 0.5 a moderate effect, and 0.8 a large effect[12].

Even though this method of analysis is considered most suitable for pooling continuous data when results are presented in different units of measurement, the issue with this method of analysis is that pooled estimates can only be interpreted as percent of non-overlap of results of two groups and not in actual change in unit of measurement [13]. As the basic purpose of this review was to give a quantitative input to LiST model in terms of actual units of measurements, we also undertook a meta-analysis to get a ‘mean difference’ by pooling results of studies that reported data in terms of mean change in weight in kilograms (kg) and mean change in length in centimeters (cm) only. In the studies where actual increase in weight and length was not given in kilograms or centimeters but that in Z score, it was back calculated with the help of software ANTHRO. In order to investigate that if this exercise introduced any bias in the results, we did sensitivity analyses for the pooled estimates (WMD) with Z scores (primary analysis) and the one with converted Z scores (secondary analysis) and reported the p-value. These estimates were used to generate recommendations for LiST model [9]. The decision about inclusion of an estimate was based on overall GRADE quality of evidence[9].More details about this method are provided in the ‘Recommendations for the LiST model’ section.

Assessment of statistical heterogeneity among the trials was done by visual inspection of forest plots, by performing the Chi^2^ (assessing the P-value) and by calculating the I^2^ statistic (calculated as I^2^ =100% x (Q-df )/Q; where Q is Cochrane’s heterogeneity statistic and df is the degree of freedom). If the P-value was less than 0.10 and I^2^ exceeded 50% and visual inspection of forest plots was indicative, heterogeneity was considered to be substantial and reasons for it were sought by doing a sensitivity analysis. If sensitivity analysis could not explain the observed heterogeneity in the pooled estimates, random effect models were used [13].This model assumes that the observed effect size from a particular study is the sum of the true effect for that study plus a normally distributed random error term, which is related to the sample size and effect size for that study and, in turn, that the true effects are themselves normally distributed[14].

## Results

### Trial flow

We identified 3795 titles from searches conducted in all databases from which 27 studies were initially considered for inclusion in the review. Five of these studies were excluded because of insufficient data to calculate change in growth parameters [15-19]. Three further studies were excluded because both intervention and the comparison group received supplementary food (e.g. fortified food vs. non-fortified food) [20-22].In the other two excluded studies; there was other co-intervention in the study group e.g. conditional cash transfer [23,24]. Altogether seventeen studies were thus included in the final review [25-41].

### Study characteristics

There were eleven studies in which intervention group received complementary food [25,26,28-30,33,34,36-38,41]. In four of these studies, provision of food was also combined with nutritional counseling to caregivers [26,34,37,38]. There were eight efficacy trials [25,26,28-30,36,37,41]while three were programs [33,34,38]. Additional File 1 presents characteristics of these studies with description of food intervention. Participants in all of the above mentioned studies were between 6-24 months of age except in three studies that included children < 6 months of age [26,29,34]. Disaggregated data for children aged > 6 months was available from two of these studies [26,29] and was used accordingly. There were a total of eight studies for the intervention of maternal education alone about complementary feeding practices [26,27,31,32,35,37,39,40]. Six of these studies were efficacy trials [26,27,31,32,35,37] while two were programs [39,40]. Additional File 2 presents the summary of educational messages given to mothers during intervention in each study. Additional File 3 presents risk of bias table for all the included studies according to Cochrane handbook [13].

### Quantitative data synthesis

#### Effect on weight gain

**Figure 2:**
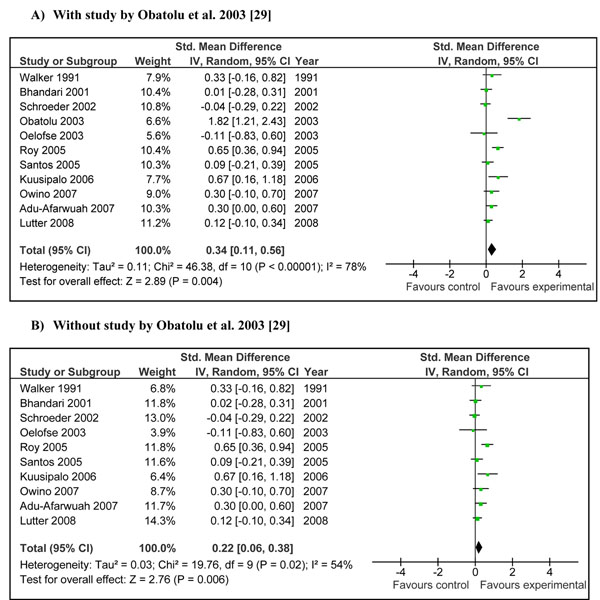
**Effect of provision of complementary food (± nutritional counseling) on weight gain: Summary estimates presented as weighed mean difference (WMD):** A) With study by Obatolu et al. 2003 [29] B) Without study by Obatolu et al. 2003 [29]

**Figure 3:**
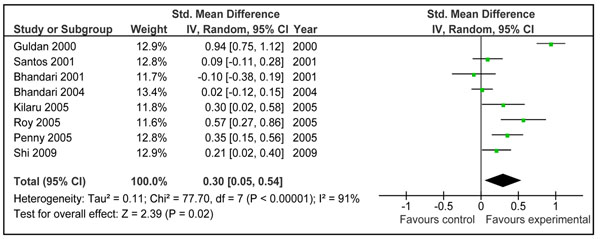
Effect of education of mothers about complementary on weight gain: Summary estimates presented as weighed mean difference

Combined results from studies where provision of complementary food (±nutritional counseling) was the main intervention showed that children in intervention group gained significantly more weight compared to control [Weighed mean difference (WMD)0.34 SD, 95 % CI 0.11-0.56, random model] (Figure 2A). The pooled estimate had a substantial heterogeneity (I^2^=78%) and one of the main contributor to the effect size and heterogeneity was study by Obatolu et al [29]. Removing this study reduced the effect size by one third and the results became relatively less heterogeneous (WMD 0.22 SD, 95 % CI 0.06-0.38, random model) (Figure 2B).Pooled estimates from studies addressing educating mothers about complementary feeding showed that this intervention also had a significant positive impact on weight gain (effect size 0.30 SD, 95 % CI 0.05-0.54, random model) (Figure 3).

#### Effect on length gain

**Figure 4:**
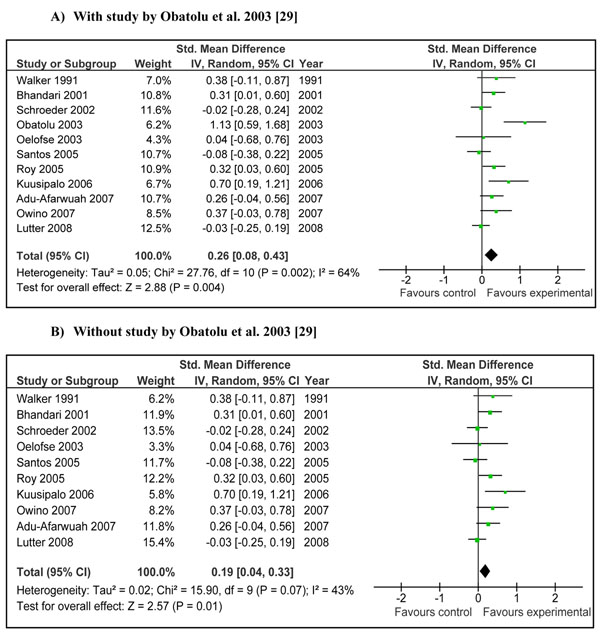
**Effect of provision of complementary food (± nutritional counseling) on height gain: Summary estimates presented as weighed mean difference:** A) With study by Obatolu et al. 2003 [29] B)Without study by Obatolu et al. 2003 [29]

**Figure 5:**
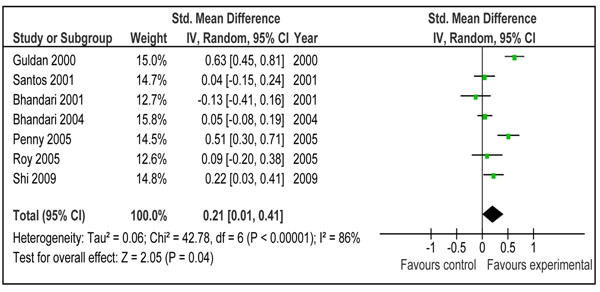
Effect of education of mothers about complementary feeding on height gain: Summary estimates presented as weighed mean difference

There was a greater increase in length in children who were provided complementary food (± nutritional counseling) compared to control (WMD 0.26 SD, 95 % CI 0.08-0.43, random model) (Figure 4A).The study by Obatolu et al. 2003 [29] was one of the major contributor to summary estimate and heterogeneity (I^2^=64%). Figure 4B showed the pooled estimate without study by Obatolu et al. [29] and we can note that both effect size and heterogeneity were reduced significantly. Educating mothers about complementary feeding also had a significant positive impact on length gain (WMD 0.21 SD, 95 % CI 0.01-0.41, random model) (Figure 5).

#### Recommendations for the LiST model

In order to give practical recommendation for LiST tool, we pooled results from studies which provided data on actual mean change in weight (kg) and mean change in length (cm). Out of eleven studies, where provision of complementary food (± nutritional counseling) was the main intervention, data on actual increase in weight (kg) and length (cm) were available from eight studies [26,28-30,33,36,38,41]. For the rest of three studies we back-calculated the change in weight (kg) and length (cm) based on the Z scores given in the study with the help of software ANTHRO.

**Figure 6:**
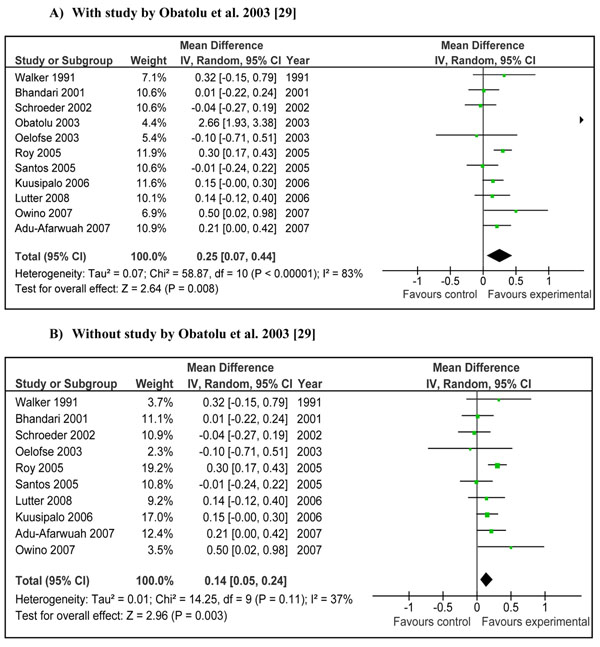
**Effect of provision of complementary food (±nutritional counseling) on weight gain (kg): Summary estimates presented as mean difference.** A) With study by Obatolu et al. 2003 [29] B) Without study by Obatolu et al. 2003 [29]

**Figure 7:**
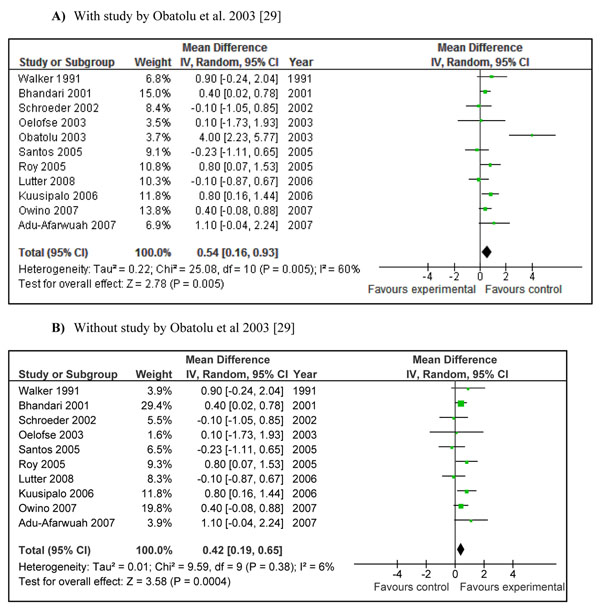
**Effect of provision of complementary food (±nutritional counseling) on height gain (cm): Summary estimates presented as mean difference** A) With study by Obatolu et al. 2003 [29] B) Without study by Obatolu et al 2003 [29]

Pooled results for *change* in weight showed that provision of complementary food (± nutritional counseling) lead to an extra gain of 0.25kg (±0.18) in the intervention group compared to control (Figure 6A). The weighted mean difference for this analysis was 0.34 SD (95 % CI 0.11-0.57, random model), which was not significantly different (p=0.96) from the primary analysis (in Figure 2A). The pooled results for increase in length for the same studies showed an extra gain of 0.54 cm (± 0.38) in the intervention group compared to control (Figure 7A). The weighed mean difference this analysis was 0.25 SD (95 % CI 0.08-0.43, random model), which was also not significantly different (p=0.98) from the primary analysis (in Figure 4A). In both of these analysis, study by Obatolu et al. 2003 [29] was an outlier and removing this study significantly reduced the effect sizes and results became less heterogeneous (Figure 6B and 7B).

**Figure 8:**
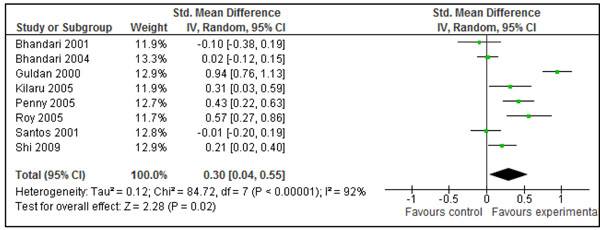
Effect of education of mothers about complementary feeding on weight gain (kg) in children: Summary estimates presented as mean difference

**Figure 9:**
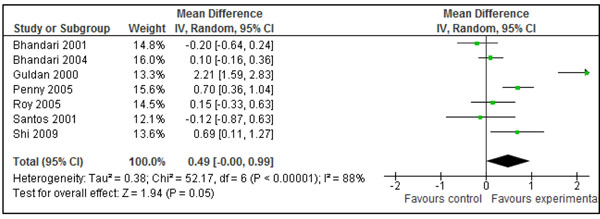
Effect of education of mothers about complementary feeding on height gain (cm) in children: Summary estimates given as mean difference

Out of eight studies, data on actual increase in weight (kg) were available from six studies addressing education of mothers about complementary feeding [26,27,31,32,35,40]. For the rest of two studies, changes in weight were back-calculated with the help of ‘Z’ scores as given in the studies [37,39]. Pooled results from all the studies showed that this intervention lead to an extra weight gain of 0.30 kg (±0.26) in intervention group compared to control (Fig 8). The WMD for this set of studies was 0.30 SD (95 % CI -0.09-0.55, random model) which was not significantly different (p=0.94) from the primary analysis (in Figure 3). Data on actual increase in length was available from five studies [26,27,31,32,35] while it was back-calculated for two studies with help of Z scores [37,39]. Combined data from these seven studies showed that this intervention leads to an extra gain of 0.49 cm (±0.50) cm in the intervention group compared to controls (Figure 9). The effect size for this set of studies was 0.19 (-0.01-0.39, random model) which was not significantly different (p=0.67) from the primary analysis (in Figure 5).

Table 1 gives the qualitative assessment of these pooled estimates according to the grade criteria [9]. The combined results for effect of provision of complementary foods (±nutritional counseling) on weight and height gain were graded as that of ‘moderate’ quality. This assessment was based on presence of significant heterogeneity and the fact that the around half of the effect size was contributed by a single study [29]. In any case, based on the available evidence, provision of appropriate complementary foods (±nutritional counseling) can increase the weight by 0.25 kg (±18) and height by 0.54 cm (± 0.38) in children 6-24 months of age. These estimates had been recommended for inclusion in the LiST model. The pooled estimate for effect of maternal education about complementary feeding on gain in weight and height were also graded as that of ‘moderate’ quality. Both of these estimates were also substantially heterogeneous. Based on available evidence, we recommend an increase of 0.30 kg (± 0.26) in weight and 0.49 cm (± 0.50) in height as effectiveness of maternal education about complementary feeding compared to control, for inclusion in the LiST model.

**Table 1:** Quality assessment of the pooled estimates of complementary feeding intervention on child growth:

Quality Assessment						Summary of findings
				Generalizability		Pooled Effect

No. of studies (ref)	Design	Limitations	Consistency	Generalizability to Population of Interest	Generalizability to intervention of Interest	Mean difference ( 95 % CI)

Effect of provision of complementary food (± education) on weight gain (kg): Quality of evidence: Moderate						

11[25,26,28-30,33,34,36-38,41]	RCT/cRCT/quasi experimental	Results are highly inflated by study by Obatolu et al. [29]. This study is also the major contributor to heterogeneity of the pooled estimate.	Heterogeneity 92 %. Random effect models used.	All the studies from developing countries	Provision of appropriate complementary food to children 6-24 months of age	0.25 (0.07-0.44) kg

Effect of provision of complementary food (± education) on height gain (cm): Quality of evidence Moderate						

11[25,26,28-30,33,34,36-38,41]	RCT//quasi experimental	Results are highly inflated by study by Obatolu et al. [29]. This study is also the major contributor to heterogeneity of the pooled estimate.	Heterogeneity 80 %. Random effect models used.	All the studies from developing countries	Provision of appropriate complementary food to children 6-24 months of age	0.54 (0.16-0.93) cm

Effect of education of mother about complementary feeding on weight gain (kg): Quality of evidence: Moderate						

8[26,27,31,32,35,37,39,40]	RCT/quasi experimental	Some of the included studies were not randomized controlled trials and it was not possible to blind the intervention in most of the studies. One of the major contributor to summary estimate and heterogeneity was study by Guldan et al. [39]. This study include mother of neonates	Heterogeneity 92 %. Random effect models used	All the studies from developing countries	Educational messages emphasized on continuity of breastfeeding, timing and frequency of complementary food, counseling on preparation of suitable food based on available local food	0.30 (0.04, 0.55)

Effect of education of mother about complementary feeding on height gain (cm): Quality of evidence: Moderate						

7[26,27,31,32,35,37,39]	RCT/quasi experimental	Some of the included studies were not randomized controlled trials and blinding assessment was not possible in most of the studies. Results for pooled estimates were not statistical significant. One of the major contributor to summary estimate and heterogeneity was study by Guldan et al. [39]. This study include mother of neonates	Heterogeneity 88 %. Random effect models used	All the studies from developing countries	Educational messages emphasized on continuity of breastfeeding, timing and frequency of complementary food, counseling on preparation of suitable food based on available local food	0.49 (-0.00-0.99)

## Discussion

Although there has been considerable progress in the development and implementation of complementary feeding practices and guidelines [42,43], relatively few reviews have quantified the effectiveness of these strategies in terms of meta-analysis. A previous review conducted for the Lancet Under-nutrition Series showed that provision of complementary food (± nutritional counseling) had a significant effect on improving linear growth (WMD 0.41, 95% CI 0.05-0.76, random model) among food insecure populations [7]. In the same review, nutritional counseling alone in food secure populations was shown to have significant effect on length (WMD 0.25, 95 % CI 0.01-0.49, random model). Dewey et al. (5) reviewed various complementary feeding strategies in depth and provided pooled effect estimates without conducting a formal meta-analysis. Education strategies for caregivers were shown to have an effect on both weight (WMD 0.28 SD, 95 % CI -0.06-0.96) and linear growth (WMD 0.20 SD, 95 % CI 0.04-0.64). Provision of complementary food (as the only treatment) and food supplements combined with nutritional counseling were also associated with positive impact on weight and linear growth[3].

Our results confirm the previously reported positive impact of complementary feeding strategies (provision of complementary food and educational strategies) on growth, however the magnitude and statistical significance of effect size differs from the above mentioned two reviews [3,7] because of differences in methods of meta-analyses. The main difference is that we pooled results for *change* in growth parameters and not that for final attained weights/heights as was done in both the above mentioned reviews [3,7]. This was to control for the possible difference in growth parameters between the two study groups at the baseline. For example in study by Bhandari et al. [26], if the results are assessed for *final* mean attained height (nutritional counseling vs. no intervention), the effect size comes to be 0.11 (95 % CI -0.18-0.39) and if we pool the results for *change* in height the effect size becomes -0.13 (95 % CI -0.41-0.16). Although both the results were statistically non-significant; seemingly positive impact in first instance was because the comparison group had less height at the baseline. These small differences in effect sizes of individual studies can affect the overall pooled estimate. Secondly, we pooled results from studies irrespective of the fact that studies reported results in Z-scores or specific unit measures. In this way more studies were included compared to studies included in the review published in Lancet Under-nutrition Series [7]. We have also added some new studies not included in the above mentioned two reviews [35,41].

Interventions in which complementary foods (±nutrition education) were provided had a significant positive impact on weight (WMD 0.34, 95 % CI 0.11-0.56) and length gain (WMD 0.26, 95 % CI 0.08-0.43) in children 6-24 months of age. The major contributor to both the above mentioned estimate was the study by Obatolu et al. [29]. The possible explanation for this was a relatively longer duration of supplementation (14 months) compared to other included studies and a smaller sample size with only 30 subjects in each arm of the study. The energy content of the supplement was also very high compared to other studies. If we exclude results of this study the heterogeneity becomes non-significant (I^2^ < 50) and effect estimates reduce to 0.22 SD (95 % CI 0.06-0.38) for weight gain and 0.19 SD (95 % CI 0.04-0.33) for height gain (Figure 2B and 4B respectively). There is also variability of results across other studies. Studies in Africa and South Asia generally showed positive effects, while those in other regions were more variable. This may be related to the relatively high prevalence of food insecurity in Africa and South Asia. If we divide the study populations into food secure (GNI >1$/day) [25,26,34,36] and food insecure (GNI < 1$/day) populations [28-30,33,37,38], the effect of provision of food seems more marked in food insecure populations compared to insecure population. The effect size for food insecure population compared to food secure population for weight gain was 0.66 vs. 0.20 and that for height gain was 0.41 vs. 0.18 (data not shown). This might indicate that provision of complementary food is more effective in promoting growth in food insecure populations compared to food secure populations especially for weight gain.

For input to Live Saved Tool, we recommended a gain of 0.25 kg (±0.18) kg in weight and 0.54 cm (± 0.38) cm in linear growth as effectiveness of provision of complementary food (± nutritional counseling). Again one of the major contributors to pooled estimate and heterogeneity was study by Obatolu et al.[29]. These limitations were incorporated in the qualitative assessment of the recommended pooled estimate by downgrading the overall quality of the pooled estimate from ‘high’ to ‘moderate’ (Table 1). The qualitative assessment of the pooled estimate was based on three components 1) the volume and consistency of the evidence; (2) the size of the effect, or risk ratio; and (3) the strength of the statistical evidence for an association between the intervention and the health outcome as reflected in the p-value [9].

The effect of education of mothers about complementary feeding on weight and height gain varied across the studies. There was substantial heterogeneity in both the pooled estimates for weight and height gain (Figure 3 and 5). The quality grade of the available evidence was assessed to be that of ‘moderate’ level (Table 1). The direction of effect was in favor of intervention in all the studies except that in study by Bhandari et al. 2001. [26]. The most likely explanation of this substantial heterogeneity was the variability of size of summary estimate across the studies (Removing study by Bhandari et al. 2001 did not affect the heterogeneity). This in turn depended on educational messages and availability of food at the baseline. If we compare results across studies, it would appear that those educational interventions had the most prominent effect in which emphasis was on feeding nutrient-rich animal-source foods (Additional File 1, Figure 3 and 5). The two studies that had the most significant effect on both weight and length gain were studies by Guldan et al. from China [39] and that of Penny et al. from Peru[31]. In both, a key message was to regularly provide an animal source food to the infant (chicken liver, egg or fish in Peru and eggs in China). The availability and utilization of these foods depends upon the economic contexts and affordability of such foods. This observation suggests that for optimal growth among infants and young children, complementary foods should have high micronutrient density from diverse food sources including animal source foods.

Given the context of food insecurity and poverty in populations with high rates of early childhood stunting, a key question pertains to the effectiveness of provision of food with nutritional counseling? Even though we did not attempt a subgroup analysis to answer this question, due to lack of adequate number of studies, we can evaluate the individual studies. Two efficacy trials where provision of food was combined with maternal nutritional counseling showed that this combination was more effective than education alone [26,37]. In first study from India [26], the food plus education group gained 250 g more weight and 0.4 cm more length than the control group during the 8-month intervention, whereas the education-only group gained only 90 g more than the control group and did not have any advantage in length gain. Similarly in study by Roy et al from Bangladesh [37], results for the education-only group were intermediate between those of the food plus education and control groups. This shows that in certain settings inclusion of a food supplement is more effective than education alone.

Our review has certain limitations. Relatively large numbers of studies had to be excluded due to non-availability of sufficient data to calculate the *change* in growth parameters (weight/height) from the baseline [15-19]. In two of these studies education approaches were evaluated [16,18] and in rest provision of complementary food (±nutritional counseling) was the main intervention [15,17,19]. Other limitations include the fact that in most of the efficacy trials blinding of assessment was not possible mainly because the study design. This might have biased the results in favor of the intervention group. Because most trials used fortified complementary foods (Additional File 1), it was not possible to determine whether the positive effects on growth were due to greater energy/protein/fat intake, greater micronutrient intake, or the combination. Finally, even though funnel plots for pooled estimates were relatively symmetrical (with one outlier i.e. study by Obatolu et al.[29]), there may be publication bias.

In conclusion, provision of complementary foods with or without nutritional counseling, has significant effect on weight and length gain especially in food insecure populations and should be recommended for the prevention of stunting in young infants and children. Educational interventions are also effective in improving complementary feeding practices and had significant effect on growth in food secure populations. Given the recognized risk factors for stunting globally [1], prevention of stunting in poor populations requires a mix of interventions that address food insecure households as a key element in reducing inequity [7]. The combination of provision of appropriate complementary foods or the resources to procure them, with nutrition education is a key intervention that should be scaled up in developing countries and the provision of specific point estimates for use in the LiST tool is a step in this direction.

## Competing interests

We do not have any financial or non-financial competing interests for this review.

## Authors' contributions

Professor Zulfiqar A Bhutta developed the review parameters and obtained support for this work. Drs Aamer Imdad and  Mohammad Yawar Yakoob undertook the literature review, data extraction and analysis under the supervision of Professor Bhutta. All contributed to the writing of the final manuscript. 

## Supplementary Material

Additional File 1 Characteristics of included studies where provision of complementary food (± Education) was the main intervention Click here for file

Additional File 2 Description of educational messages given to mothers Click here for file

Additional File 3 Risk of bias table for included studies according to the latest Cochrane handbook Click here for file
